# Macrophage-Specific Chemokines Induced via Innate Immunity by Amino Acid Copolymers and Their Role in EAE

**DOI:** 10.1371/journal.pone.0026274

**Published:** 2011-12-15

**Authors:** Joseph Kovalchin, Jeffrey Krieger, Michelle Genova, Norio Kawamoto, Michael Augustyniak, Kathryn Collins, Troy Bloom, Allyson Masci, Tara Hittinger, Ingrid Dufour, Jack L. Strominger, Eric Zanelli

**Affiliations:** 1 Peptimmune, Inc., Cambridge, Massachusetts, United States of America; 2 Department of Stem Cell and Regenerative Biology, Harvard University, Cambridge, Massachusetts, United States of America; Massachusetts General Hospital and Harvard Medical School, United States of America

## Abstract

The random amino acid copolymer poly(Y,E,A,K)_n_ (Copaxone®) is widely used in multiple sclerosis treatment and a second generation copolymer poly(Y,F,A,K)_n_ with enhanced efficacy in experimental autoimmune encephalomyelitis in mice has been described. A major mechanism through which copolymers function to ameliorate disease is the generation of immunosuppressive IL-10-secreting regulatory T cells entering the CNS. In addition, the antigen presenting cell to which these copolymers bind through MHC Class II proteins may have an important role. Here, both CCL22 (a Th2 cell chemoattractant) in large amounts and CXCL13 in much smaller amounts are shown to be secreted after administration of YFAK to mice and to a smaller extent by YEAK parallel to their serum concentrations. Moreover, bone marrow-derived macrophages secrete CCL22 *in vitro* in response to YFAK and to higher concentrations of YEAK. Strikingly, these chemokines are also secreted into serum of MHC Class II −/− mice, indicating that an innate immune receptor on these cells also has an important role. Thus, both the innate and the adaptive immune systems are involved in the mechanism of EAE amelioration by YFAK. The enhanced ability of YFAK to stimulate the innate immune system may account for its enhanced efficacy in EAE treatment.

## Introduction

Two random amino acid copolymers have been described, the administration of which ameliorates experimental autoimmune encephalomyelitis (EAE) and several other autoimmune diseases in mice and other rodents using several different models. They are poly(Y,E,A,K)_n_ (called YEAK, Copaxone®, glatiramer acetate, Copolymer-1) [1], [2] and poly(Y,F,A,K)_n_ (called YFAK) [3]. In these models, YFAK is far more effective than YEAK [4], [5], [6], [7]. YEAK (Copaxone®) has been in clinical use for several decades for the treatment of multiple sclerosis (MS) although its usefulness in this disease is limited to a ∼30% reduction in frequency of relapses. YFAK has gone through Phase Ia and Ib clinical trials (the latter in patients with secondary progressive MS) with no sign of toxicity [8], [9] and is ready for a Phase II trial in patients with relapsing, remitting MS.

Both copolymers have been thought to exert their primary immunosuppressive action through the generation of immunosuppressive T cells that secrete IL-10 as a major immunomodulatory cytokine, as well as other cytokines [6]. Over the years, however, a number of papers have appeared indicating that the antigen presenting cell, defined as either a dendritic cell or a macrophage, is also modified by copolymer treatment and that it plays an important role in the disease process [10]–[13]. In addition, IL-10 secreting B cells have been reported to be produced as a result of YEAK treatment [14].

The purpose of the present study was to define further the nature of the antigen presenting cell modified by copolymer treatment and its relationship to the IL-10 secreting T cells that have been described previously.

## Methods

### Amino Acid Copolymers YFAK and YEAK

YFAK is a mixture of random-sequence peptides composed of the amino acids L-tyrosine, L-phenylalanine, L-alanine, and L-lysine in the approximate molar ratios of 1.0∶ 1.3∶ 24.0∶ 6.0, respectively. YFAK is manufactured by solid phase synthesis on pre-loaded Wang resin with base labile Fmoc-groups. The excess amino acid derivatives and coupling reagents are removed by filtration. YFAK is cleaved, N-acetylated, precipitated, washed and dried under vacuum. YEAK (Copaxone®, purchased from Hanna Pharmaceuticals (Wilmington, DE)) was stored at 4°C at a concentration of 20 mg/mL according to the manufacturer's package insert. YFAK or YEAK was diluted in 42 mg/mL mannitol (Sigma, St. Louis, MO) in water at concentrations indicated. Compounds were administered s.c. (subcutaneously) interscapularly at a dose volume of 100 µL/10 g body weight.

### Pharmacokinetic Assays for YFAK and YEAK

All mouse work herein discussed was performed under an approved protocol with the review of Harvard University's Standing Committee on the Use of Animals in Research and Teaching, under the Guidelines for the Use of Vertebrate Animals in Research and Teaching of the Faculty of Arts and Sci-ences of Harvard University, and under the NIH Guide for the Care and Use of Laboratory Animals. Ameliorative steps were taken whenever animals were injected or observed in disease states including the administration of anesthesia, food supplementation, and temperature modification. The HU/FAS animal care and use program maintains full AAALAC accreditation, is assured with OLAW (A3593-01), and is currently registered with the USDA. This work was carried out under Protocol 99-01, ap-proved in latest amendment by IACUC on 05/13/11.

The pharmacokinetic (PK) assays (validated in CD-1 male mice obtained from Avogadro, Fontenilles, France and bred at Charles River Laboratories, Wilmington, MA) for YFAK and YEAK are direct competition ELISAs [8]. Briefly, YFAK or YEAK are immobilized on a 96-well microtiter plate overnight at 4°C, then blocked for two hours with 300 µL per well of PBS/10% FBS, and washed three times with 300 µL per well of PBS/0.05% Tween 20 using a plate washer. Mouse serum containing known or unknown concentrations of YFAK/YEAK were added to the washed plates along with purified biotinylated anti-YFAK/YEAK antibodies and Protein A and incubated for 2 hours on a plate shaker. Unbound material was washed away and diluted streptavidin-HRP conjugate added to the wells and incubated for 1 hour. After washing, substrate (TMB from BD Biosciences–Pharmingen, San Diego, CA) was added and circulation continued for 15 minutes, yielding a blue color that turns yellow when stop solution (2N H_2_SO_4_) is added. The optical density was measured at 450 nm, and a standard curve generated. The intensity of the color measured is proportional to the amount of biotinylated anti-YFAK/YEAK antibody bound by the immobilized YFAK/YEAK.

YFAK/YEAK biotinylated antibodies were Protein A purified from rabbit polyclonal antiserum generated against the appropriate antigen. Briefly, rabbits were immunized with YFAK or YEAK. The rabbit polyclonal antiserum was diluted 1∶1 with pH 8.0 buffer, added to a Protein A column (column was equilibrated with the pH 8.0 buffer), incubated one hour at room temperature, then the unbound proteins were washed off the column with the pH 8.0 buffer. The specific antibody was eluted with pH 2.8 buffer, dialyzed against PBS at 4°C overnight and the protein concentration was determined using Coomassie blue. The dialyzed antibody was then incubated two hours at room temperature with a ten molar excess of biotin and dialyzed against PBS at 4°C overnight. The dialyzed biotinylated antibody was concentrated using a desalting column and the protein concentration was determined using Coomassie. The mole-to-mole ratio of biotin to protein was determined using the HABA method (Pierce Biotechnology, Rockford, IL).

### *In Vivo* Serum/Plasma Collection

Male CD-1 mice (Charles River Laboratories) at 8–12 weeks of age, female SJL mice (Charles River Laboratories) at 7–9 weeks of age, and MHC II −/− mice (B6.SJL(129)-*Ptprc^a^*/BoyAiTac H2-Ab1^tm1Gru^ N7+N6) and their wild type control littermates (Taconic Farms, Hudson, NY) at 8–11 weeks of age were used for PK and chemokine release experiments. Mice were dosed s.c. interscapularly as indicated using a 27 gauge needle for each experiment. Whole blood was collected by cardiac puncture with a 25 gauge needle and 1 mL syringe into tubes which do not contain anti-coagulant (yellow topped Vacutainer® tubes with serum separator). The blood sample was allowed to clot at room temperature for 15–30 minutes and then was centrifuged at 4°C, 10,000 RPM, for 10 minutes in order to isolate serum. Whole blood was also collected into tubes containing anti-coagulant (purple topped Vacutainer® tubes with EDTA) for plasma preparation. Blood was collected, inverted several times, and then centrifuged at 4°C, 10,000 RPM, for 10 minutes. Both serum and plasma were collected, placed on dry ice, and then stored at −80°C until further analysis.

### *In Vitro* Bioassay Using Cells of the RAW 264.7 Macrophage Cell Line

RAW264.7 cells (ATCC, Manassas, VA) were plated in 96 well U-bottom tissue culture plates at 5×10^5^ cells per well with and without compounds incubated in 200 µl culture medium (10%FBS (Thermo Scientific-Hyclone, Waltham, MA) / DMEM with 1%PSG (Invitrogen-Gibco, Carlsbad, CA)) for 24 hours at 37°C with 5% CO_2_ in a humidified environment. Cell-free culture supernatant was collected and immediately frozen at −80°C for future testing with a commercial CCL22 ELISA kit following manufacturer's protocol (R&D Systems, Minneapolis, MN).

### *In Vitro* Bone Marrow Cultures

Femurs were excised and bone marrow cells were collected from twenty-five naïve female SJL mice (Charles River Laboratories; 9–12 weeks of age) by snipping off the ends and flushing the marrow with HL-1 media (Lonza-Biowhittaker, Walkersville, MD) using a 1 mL syringe equipped with a 30 gauge needle. Single-cell suspensions from femur-derived bone marrow cells were depleted of T and B cells by negative selection using a combination of CD90.2 and CD19 magnetic beads with the method described in the manufacturer's package insert (Miltenyi Biotec, Auburn, CA). The residual cells, which are enriched for myeloid progenitors, were re-suspended and plated at 1×10^6^ cells per well to 24 well tissue culture plates in 2 mL CM^+^ media, defined as HL-1 media containing 10 µM HEPES (Invitrogen-Gibco), 1 mM Sodium Pyruvate (Invitrogen-Gibco), 2 mM L-glutamine (Invitrogen-Gibco), 1% Penicillin/Streptomycin/L-glutamine (Invitrogen-Gibco), 1% nonessential amino acids (NEAA) (Invitrogen-Gibco), and 50 µM 2-Mercaptoethanol (Sigma)) supplemented with 10% FBS (Thermo Scientific-Hyclone), 10 ng/mL IL-3 (R&D Systems), and 2.5 ng/mL TNF-α (R&D Systems) to obtain bone marrow –derived macrophages [18] in addition to equimolar concentrations (1.5, 3, 6, and 12 µM) of YFAK, YEAK, or the encephalatogenic peptide PLP139–151 (A&A Labs). The cells were cultured for six days in a humidified 37°C incubator with 5% CO_2_. On day 7, half of the medium was removed from each well and the cells were then treated with an equivalent volume of LPS (Sigma, St. Louis, MO) at a final concentration 1 µg/mL, without additional YFAK, YEAK, or PLP139–151 peptide, for two days. On study day 9, cell-free culture supernatants were harvested, aliquoted, and immediately frozen at −80°C. After collection of supernatants, the ends of 1.8 cm blade cell scrapers (BD Falcon, San Jose, CA) were trimmed to fit into the wells of the 24 well tissue culture plates, and cells from *in vitro* bone marrow cultures were collected by scraping. Cell viability was tested using trypan blue exclusion. Cell viability was shown to be 92.1±0.5% (mean ± SEM) (data not shown).

### Flow Cytometric Analysis of *In Vitro* Bone Marrow Cultures

Bone marrow cells were analyzed by flow cytometry on day 1, both pre- and post- depletion, and on day 9. At each day, cells were washed twice in staining buffer containing 2% FBS and 0.09% sodium azide at pH 7.4 (BD Pharmingen, San Diego, CA). To decrease non-specific cell staining, Fc receptors were blocked by incubating cells for 5 minutes on ice with an optimal concentration of rat anti-mouse CD16/CD32 (BD Pharmingen) diluted in staining buffer. Cells were then stained for 30 minutes on ice in the dark by the addition of an antibody conjugate panel selected to enable the phenotypic analysis of monocytes and to confirm the absence of T and B cells (F4/80: FITC- eBioscience, San Diego, CA, and the remainder from BD Pharmingen: B220: PE-, CD11b: PerCP-Cy5.5-, CD11c: PE-Cy7-, CD3: APC-, and GR-1: APC-Cy7). After staining, cells were washed twice with staining buffer, then red blood cells (RBC) were lysed and cells were fixed (FACS Lyse, BD Pharmingen) on day 1, or cells were fixed without lysis (CytoFix, BD Pharmingen) on day 9. Following 2 washes with staining buffer, cells were resuspended to staining buffer and acquired on a Becton Dickinson FACS CANTO II flow cytometer with FACSDiva software.

### Evaluation of Cytokines and Chemokines From *In Vitro* Bone Marrow Cultures and *In Vivo* Assays

Cell-free culture supernatants, EDTA plasma, and serum samples were sent to Rules-Based Medicine (Austin, TX) for analysis of cytokine and chemokine production using their Rodent Multi-Analyte Profiling (MAP) Version 1.6. EDTA plasma samples were also tested for CCL22 and CXCL13 and the cell-free culture supernatants for CCL22, CXCL13, and TNF-α using commercially available ELISA kits following manufacturer's protocol (R&D Systems). For IL-3 analysis, female SJL mice were dosed daily for 5 days with 0.25, 2.5, or 25 mg/kg of YFAK or YEAK. Spleens were collected after a one week resting period. Splenocytes were re-stimulated for 3 days with 5 µg/mL of corresponding copolymer after which cell culture supernatants were harvested and tested for IL-3 secretion using commercially available ELISA kits following the manufacturer's protocol (R&D Systems).

## Results

### Copolymer plasma levels and release of the macrophage-specific chemokines CCL22 and CXCL13 *in vivo* induced by copolymers

**Figure 1:**
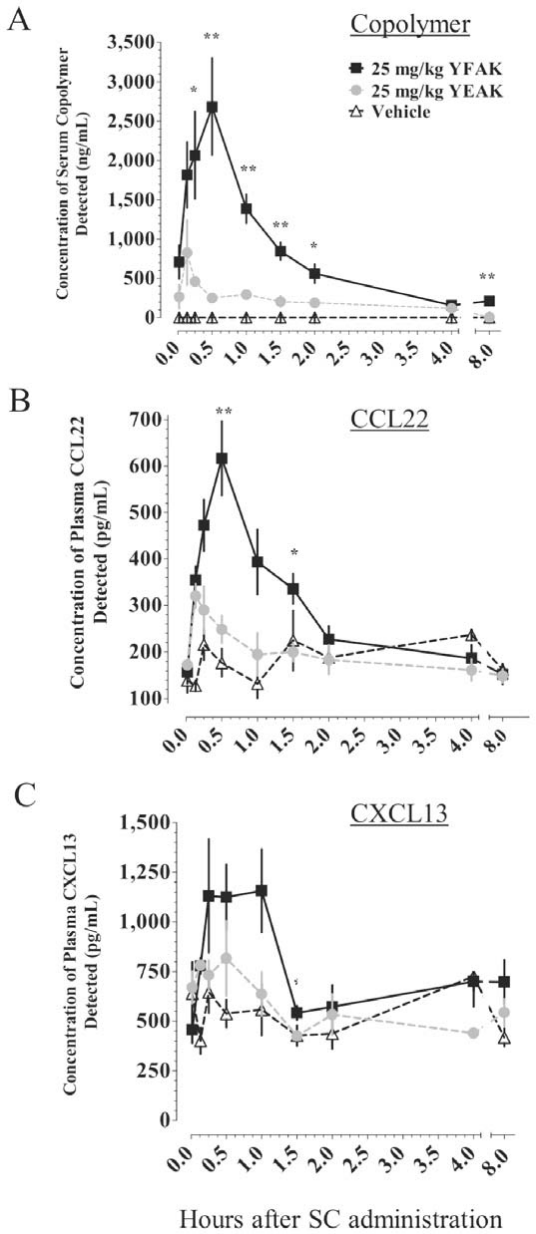
Kinetics of serum copolymer and of plasma chemokine levels after administration of YFAK or YEAK. Data are shown as mean ± SEM from an experiment in which 3–4 mice were euthanized and samples collected at each time point. (A) Serum YFAK (ng/mL) is significantly greater than YEAK (ng/mL) at multiple time points from 15 to 120 minutes. (B, C) YFAK induced increased plasma CCL22 (pg/mL) and CXCL13 (pg/mL) levels when compared to YEAK as shown. Significance was calculated using an unpaired t-test: YFAK vs. YEAK or Control * p≤0.05, ** p≤0.01.

Recently, a role for myeloid cells, as well as IL-10-secreting T cells, in the protective response to amino acid copolymers has been demonstrated as the ability of YEAK to stimulate macrophages, termed “M2 regulatory macrophages,” that on adaptive transfer decreased disease severity [13]. To address this question further and to define the nature of the macrophages produced, first pharmacokinetic (PK) assays to quantify YFAK or YEAK in serum were developed [8]. Assays for the presence of these copolymers in serum were carried out in CD-1 male mice because they are larger (∼35 grams) than SJL mice (∼20 grams), making it possible to obtain sufficient serum/plasma from a single mouse to test both for copolymers and for substances released in the same sample. Male CD-1 mice were dosed once s.c. with Vehicle (42 mg/L mannitol in H_2_O), 25 mg/kg YFAK in mannitol, or 25 mg/kg YEAK in mannitol. Serum concentrations over an 8-hour time course were analyzed (Figure 1A). The high dose of 25 mg/kg was used in order to amplify the response, allowing the kinetics of the copolymer to be followed. Serum concentrations of YFAK were significantly greater than those of YEAK for all time points between 15 and 120 minutes post administration. Mice receiving YFAK peaked at 2,680±613 ng/mL at 30 minutes while those receiving YEAK peaked at only 826±414 ng/mL at 8 minutes. Thus, YFAK reached a higher C_max_ slower than YEAK and remained in circulation much longer than YEAK. Additional PK profiles showed that a significant linear correlation, tested in the range of 0–≥80 mg/kg amino acid copolymers, existed between the s.c. dose administered and the mean serum C_max_. At the low dose of 2.5 mg/kg (50 µg to a 20 g mouse), the efficacious dose in EAE, YFAK was at or below the level of detection for the current PK assay. A significant linear correlation was also found between the s.c. dose and the total systemic exposure, as defined by the area under the curve (AUC) for the serum concentration of YFAK and YEAK for the entire time course. The PK assay that was developed is sensitive enough not only to identify the full length YFAK (52 a.a., MW 4500) or YEAK (∼70 a.a. average, MW 7500) but also to detect sub-fragments (≥30mers) which may occur as a result of peptidase digestion and may be biologically active.

Evidence of macrophage activation through the release of M2 chemokines [15] was then sought. Within minutes after s.c. administration and detection of the copolymer in plasma, in parallel two substances appeared prominently in mouse plasma following *in vivo* administration of both copolymers, viz. CCL22 (originally called MDC, macrophage-derived chemokine) and CXCL13 (BLC, B lymphocyte chemokine). Greater transient increases in CCL22 and CXCL13 plasma concentrations were observed in mice dosed with YFAK as compared to those dosed with YEAK. Mice receiving YFAK reached a peak of 617±80 pg/mL for plasma CCL22 at 30 minutes compared to mice receiving YEAK that peaked at 320±26 pg/mL of CCL22 at 8 minutes (Figure 1B). Increased CXCL13 plasma concentrations were also detected within minutes after YFAK administration s.c. (Figure 1C), reaching a maximum plasma concentration of 1,160±210 pg/mL at 60 minutes post administration. Plasma CXCL13 in mice dosed with YEAK peaked at 816±188 pg/mL at 30 minutes but was not significantly greater than the vehicle control. A significant linear correlation existed between the serum concentration of YFAK and the plasma concentration of CCL22 and CXCL13. Similar, though less complete, data were also obtained in SJL mice (data not shown).

#### Role of innate immune system in chemokine release

**Figure 2:**
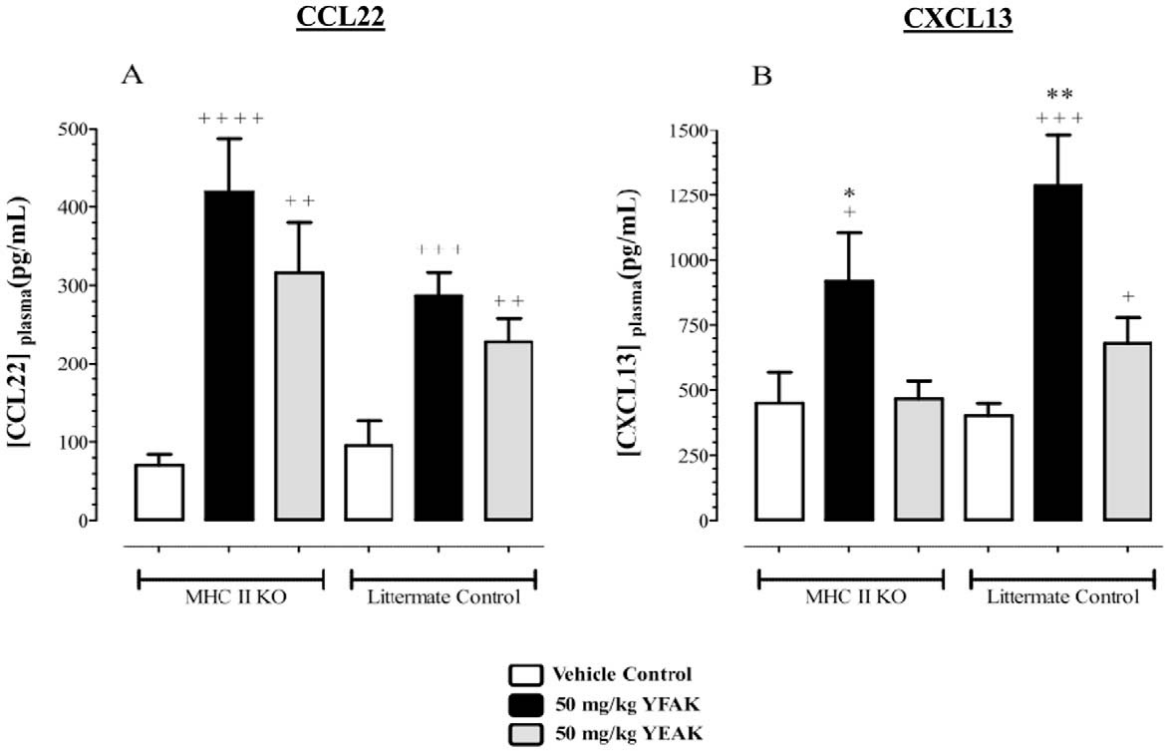
Copolymer-mediated induction of CCL22 and CXCL13 in MHC Class II KO mice. Female MHC Class II deficient mice and their littermate controls (n = 9–10) were administered one subcutaneous dose of 50 mg/kg YFAK, 50 mg/kg YEAK, or Vehicle Control. Blood was collected and plasma was prepared 30 minutes post administration and tested for CCL22 (A) and CXCL13 (B). Data is shown as mean (pg/mL) ± SEM. Significance was calculated using an unpaired t-test: YFAK or YEAK vs. Vehicle Control + p≤0.05; + + p≤0.01; + + + p≤0.001; + + + + p≤0.0001; YFAK vs. YEAK * p≤0.05; ** p≤0.01.

Since binding of amino acid copolymers to MHC class II molecules has been considered to be central to their mechanism of action [16], [17], it was important to investigate whether or not the induced release of chemokines by YFAK or YEAK was a MHC Class II protein (MHC II) dependent response. However, no differences in chemokine release into plasma were observed in MHC II deficient mice (I-Aβ knockout in the B6/SJL background that does not express I-E, see Methods) compared to B6/SJL congenic controls. YFAK and YEAK significantly increased CCL22 production compared to vehicle treatment in both strains of mice (Figure 2A). YFAK also significantly increased CXCL13 production in both strains of mice while again YEAK had no effect on CXCL13 plasma level in either strain (Figure 2B). The release of CCL22 and CXCL13 by YFAK or YEAK was *independent* of MHC II. Thus, an innate immune receptor(s) present on myeloid cells is (are) capable of interacting with YFAK and YEAK to mediate the secretion of these chemokines.

#### Secretion of CCL22 from RAW 264.7 Cells Induced by the YFAK and YEAK Copolymers and by YFAK Oligomers

**Figure 3:**
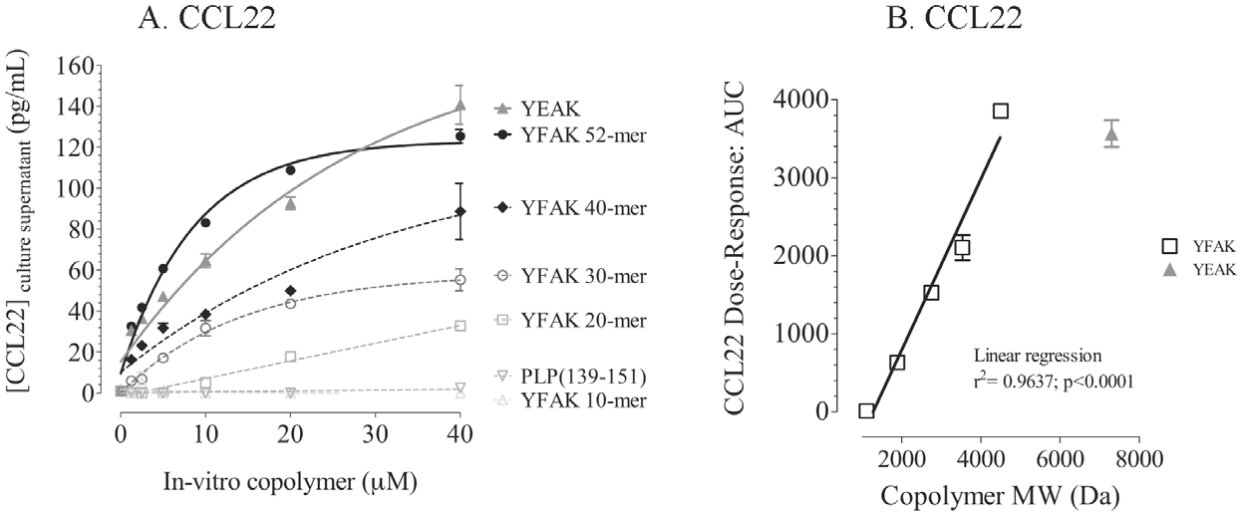
CCL22 secretion induced from the RAW 264.7 macrophage cell line. (A) RAW264.7 cells were plated on 96 well U-bottom tissue culture plates at 5×105 cells per well and incubated in culture medium (10% FBS/DMEM with 1% PSG) without or with molar equivalent concentrations of copolymers for 24 hours at 37°C with 5% CO2 in triplicate. Cell-free culture supernatant was collected and immediately frozen at −80°C for testing using a commercial CCL22 ELISA kit. Data are represented as a non-linear regression curve fit and shown as mean (pg/mL) ± SEM. (B) Linear correlation be-tween area under the curve of CCL22 production and the length/molecular weight (Da) in the culture supernatants of RAW264.7 cells incubated in culture medium with YEAK, YFAK, or molar equivalent of truncated YFAK (r2 = 0.9637, p<0.0001.

A similar phenomenon was observed *in vitro* when YFAK was tested on the differentiated mouse macrophage line RAW 264.7. These cells secreted CCL22 in increasing amounts at increasing concentrations of copolymers, reaching a plateau at ∼20 µM YFAK (MW 4500) or ∼40 µM YFAK (MW 7300) (Figure 3A). The availability of an unlimited number of cells of this cell line also made possible examination of the effects of copolymer size on the release of CCL22. Truncated fragments of the YFAK 52-mer (MW 4,500), 10mer (MW 1,114), 20mer (MW 1,890), 30mer (MW 2,751), and 40mer (MW 3,534) were isolated during the course of copolymer manufacturing. The release of CCL22 was essentially linear with the length (Figure 3B). Neither the copolymers nor LPS induced secretion of CXCL13 from RAW 264.7 cells.

#### Chemokine and Cytokine Production *In Vitro* by Bone Marrow Derived Cells

Next, bone marrow derived myeloid cells [15], [18] were employed to analyze *in vitro* the secretion of the two monocyte-derived chemokines CCL22 and CXCL13 associated with immunoregulation. In brief, myeloid progenitors prepared from bone marrow cells of SJL mice (see Methods) were incubated for six days in medium containing 10 ng/ml IL-3, 2.5 ng/ml TNFα, and equimolar concentrations of YFAK or YEAK at 0, 1.5, 3, 6, and 12 µM followed by 2 further days incubation with 1 µg/ml LPS. IL-3 has been shown to induce M2 macrophage differentiation in bone marrow myeloid progenitors [18]. These concentrations of copolymers are in the range of those that would be obtained *in vivo* after the administration of the therapeutic dose of 50 µg to SJL mice. Phenotyping of the resulting myeloid cell population indicated a marked increase of CD11b+ F4/80^neg-low^ cells with a corresponding decrease in CD11b^+^ F4/80^high^ cells in mice treated with YFAK (Figure S1). Similarly, the frequency of CD11b^+^ Gr-1^high^ cells increased while that of CD11b^+^ Gr-1^mid^ decreased in the myeloid cells of YFAK-treated mice (Figure S2). No change in either cell population was found in control PLP 139–151-treated mice or in those treated with YEAK. CD11b^+^F4/80^neg-low^ Gr-1^high^ cells seen after treatment with YFAK are phenotypically an immature macrophage population, possibly in the myeloid suppressor lineage [19].

**Figure 4:**
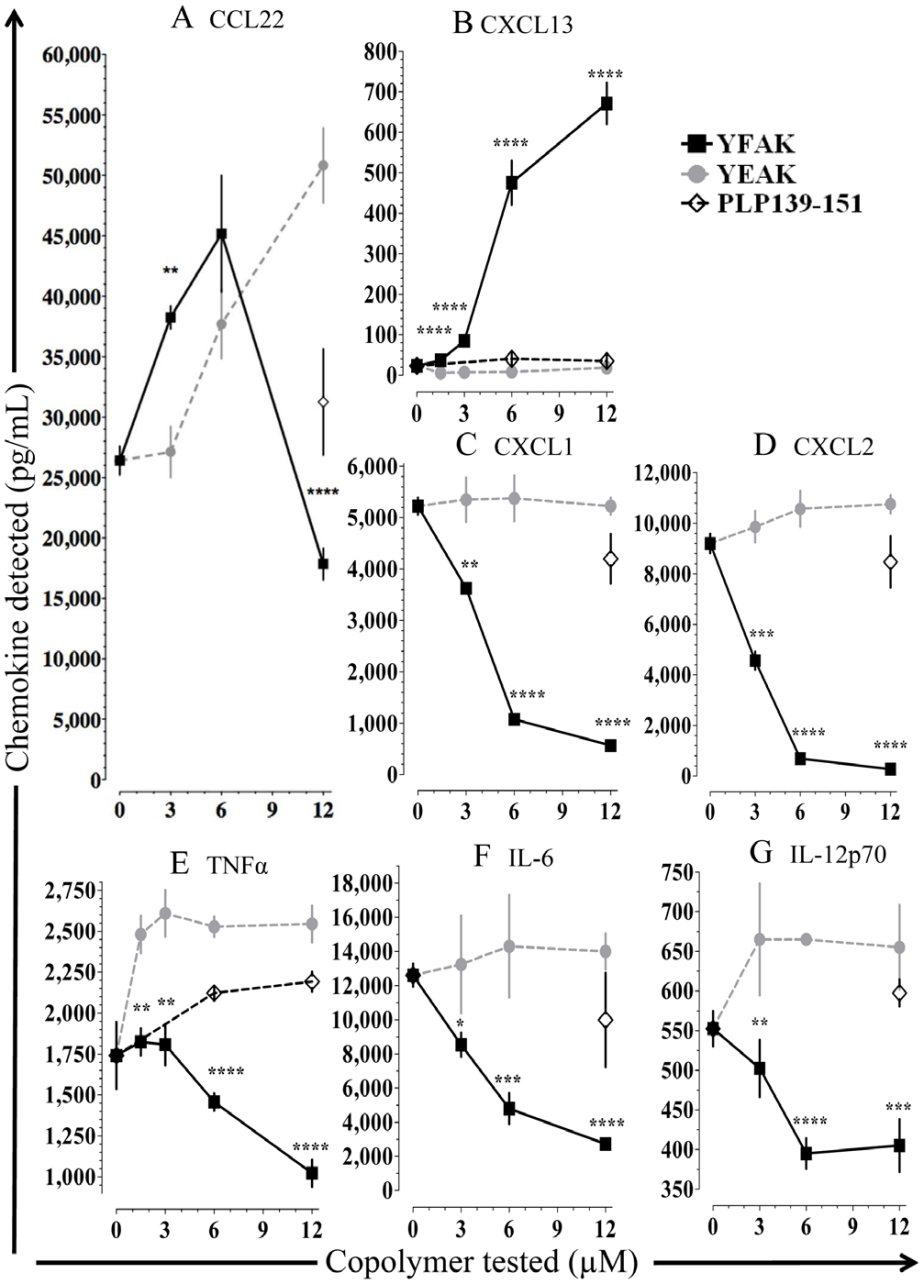
Effects of YFAK and YEAK on chemokine and cytokine secretion from bone marrow-derived myeloid cells. Cytokine and chemokine production was analyzed from bone marrow-derived myeloid cells (BMMC). Data are from a representative experiment in which samples were run in 4 replicates and are shown as mean (pg/mL) ± SEM. In vitro compound concentrations administered were 1.5, 3.0, 6.0, and 12.0 µM. Significance was calculated using an unpaired t-test: YFAK vs. YEAK * p≤0.05, ** p≤0.01, *** p≤0.001, **** p≤0.0001.

These myeloid cells secreted a very high baseline level of CCL22 (26,400 pg/ml), but incubation with YFAK, and less effectively YEAK, induced much higher dose dependent levels peaking at 45,200 pg/ml for YFAK at 6 µM and at 52,500 pg/ml for YEAK at 12 µM (Figure 4A). The decrease of CCL22 as seen at 12 µM was paralleled by a decrease of IL-4 and IL-13 secretion by T cells at this extremely high level in clinical studies [9], and has also been observed in additional studies of myeloid cells differentiated in the presence of GM-CSF that secrete CCL22 upon stimulation with YFAK (N.K., unpublished observation).

After treatment of the myeloid cells with YFAK, the concentration of CXCL13 secreted increased to 671±51 pg/mL, while treatment with YEAK or PLP139–151 resulted in no change from the baseline level of ∼20 pg/mL (Figure 4B).

The effects of YFAK and YEAK on supernatant concentration of CXCL1 and CXCL2 that are associated with neutrophil chemotaxis were also examined. Reciprocally, the concentration of CXCL1 and CXCL2 fell from baseline levels of 5,225±165 pg/mL or 9,193±385 pg/mL respectively to near 0 in both cases while little or no change was observed with YEAK or PLP139–151 (Figure 4C and D).

The concentration of the pro-inflammatory cytokines TNF-α, IL-6, and IL12p70 were all substantially decreased by YFAK, but not by YEAK or PLP139–151 (Figure 4E, F, G). This latter observation contrasts with previous work demonstrating the capacity of YEAK to inhibit TNF-α production by THP-1 macrophages stimulated with LPS and IFN-γ [20]. PLP 139–151 had little or no effect on concentrations of IL-6 or IL-12p70 and slightly increased the concentrations of TNF-α (Figure 4E, F, G).

These assays were carried out either using the Rules-Based Medicine Rodent MAP version 1.6 panel that examines 69 secreted factors (Table S1, www.rulesbasedmedicine.com) or by ELISA. No significant secretion beyond that of the medium controls was observed for any of the other chemokines or cytokines in the panel on stimulation with YFAK, YEAK, or PLP139–151 (an additional control) in the range of 1.5–12 µM (with the exception of CCL9 – see Discussion). Notable was the absence of induced secretion of IL-10 from the myeloid cells beyond the low level of 1270 pg/ml found in the medium control.

#### Macrophage-stimulating IL-3 Secretion From Splenocytes of Mice Treated with YFAK or YEAK

Since both YFAK and YEAK stimulate splenocytes and spleen-derived T cell lines to secrete T_H_2 cytokines including IL-10, IL-13, and IL-4, the latter two of which have been reported to induce M2 macrophage differentiation [15], the question arose whether the macrophage-stimulating IL-3, a T cell cytokine, was also produced. Moreover, this cytokine had been added to the culture conditions used to differentiate bone-marrow cells into macrophages in the presence of YEAK or YFAK (Figure 4 and Figure S1, S2).

**Figure 5:**
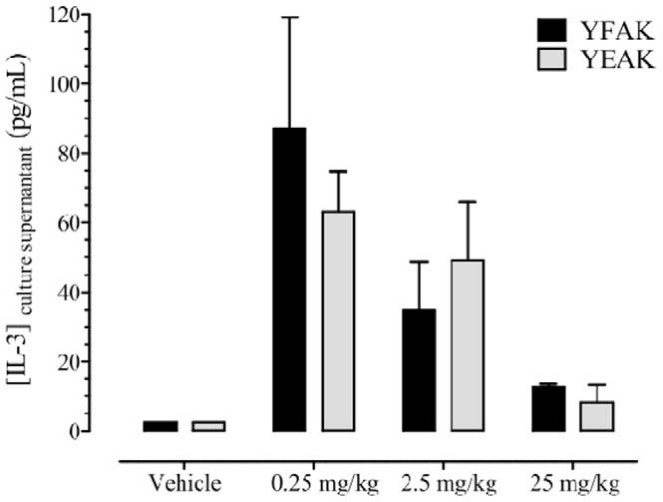
IL-3 secretion induced by YFAK and YEAK from splenocytes. Female SJL mice were dosed daily for 5 days with 0.25, 2.5, or 25 mg/kg of YFAK or YEAK. Spleens were collected after a one week resting period. Splenocytes were re-stimulated for 3 days with 5 µg/mL copolymer. Splenocytes harvested from Vehicle-treated mice and stimulated with YFAK or YEAK had no detectable level of IL-3. Mean (pg/mL) ± SEM of IL-3 is shown. No significant difference in the release of IL-3 in splenocytes re-stimulated with YFAK or YEAK was evident.

To answer this question, female SJL mice were dosed daily for 5 days with 0.25, 2.5, or 25 mg/kg of YFAK or YEAK. Spleens were collected after a one week resting period. Splenocytes were re-stimulated for 3 days with 5 µg/mL of corresponding copolymer after which cell culture supernatants were harvested and tested for IL-3 secretion. Splenocytes from mice treated with 0.25 mg/kg of YEAK or YFAK produced the greatest concentrations of IL-3 (Figure 5). As the dose of YFAK or YEAK increased, levels of IL-3 decreased. No significant difference in the release of IL-3 in splenocytes stimulated with comparable doses of YFAK or YEAK was observed.

## Discussion

Since the approval of the random amino acid copolymer YEAK (Copaxone®) for the treatment of RR-MS, YFAK, a second generation copolymer with enhanced efficacy in mouse models of EAE has been described. Understanding the mechanism of action of the copolymers has engaged numerous laboratories (reviewed by [13], [21]–[22]). Here, YFAK and YEAK are shown to have dramatically different effects on the immune system in mice, especially with respect to effects on the innate immune response that plays an important role in efficacy. The major molecular differences between YFAK and YEAK are highlighted in Table 1. YFAK is a solid phase synthesized acetylated 52mer random copolymer (relative ratio: Tyrosine 1.0, Phenylalanine 1.2, Alanine 23.5, and Lysine 6.0) that has a strong net positive charge. YEAK, on the other hand, is a solution phase synthesized non-acetylated 20–200mer random copolymer (relative ratio: Tyrosine 3.5, Glutamic Acid 1.5, Alanine 4.5, and Lysine 3.6) that has a slightly net positive charge. YFAK is significantly more efficacious than YEAK in the prophylaxis of the induction of EAE or in the treatment of established EAE in mice ([4], [5], and further documented in more detail in Figure S3).

**Table 1:** Molecular Properties of YFAK and YEAK.

	YFAK	YEAK
**Synthesis**	Solid Phase	Solution Phase
**Amino Acid Input**	YFAK (Tyrosine 1.0, Phenylalanine 1.2, Alanine 23.5, Lysine 6.0)	YEAK (Tyrosine 1.0, Glutamic Acid 1.5, Alanine 4.5, Lysine 3.6)
**Peptide Charge**	Strong Net Positive Charge	Slightly Net Positive Charge
**Molecular Weight Distribution**	4–5 kDa	5–9 kDa
**Peptide Length**	52-mer	20–200-mer
**Acetylation**	N-terminus	No

The serum levels reached in mice after s.c. administration (measured using a newly developed antibody-based method) of YFAK and its duration were much larger than those for YEAK (Figure 1). In spite of its peptidic nature, YFAK (and sub-fragments ≥30-mers) were detectable in mouse serum for several hours. YFAK may be binding to a plasma component that prevents its rapid degradation by peptidases, possibly a function of its strong net positive charge when compared to YEAK. Acetylation of YFAK on the N-terminus also may help protect it from peptidase degradation. A significant linear correlation was observed between the mean serum concentration of YFAK or YEAK (C_max_) and the administered s.c. dose.

The detection of YFAK and YEAK in the serum coincided with the appearance in plasma of CCL22 and CXCL13 within minutes of copolymer administration (Figure 1). These chemokines are known to be secreted by regulatory M2a and M2c macrophages, respectively [15], also known as alternatively activated macrophages [23]. YFAK induced a significantly higher C_max_ for CCL22 and CXCL13 than YEAK. Production of CCL22 and CXCL13 induced by both copolymers was independent of MHC class II as shown by their secretion in the MHC Class II−/− mice (Figure 2). A second innate immune receptor for these peptide copolymers, in addition to MHC Class II, must be functional. Systemic exposure to copolymers may be important to insure a broad effect on the innate immune system. The enhanced efficacy of YFAK in mice may be related to its intrinsic action on myeloid cells and the enhanced time of serum availability. Although the effects of YFAK and YEAK on plasma CCL22 and CXCL13 during induction of EAE and its therapy was not measured in these experiments, the analysis of serum CCL22 during administration of YFAK to patients with secondary progressive multiple sclerosis in a Phase Ib clinical trial was carried out and the appropriate elevation was observed [9].

**Figure 6:**
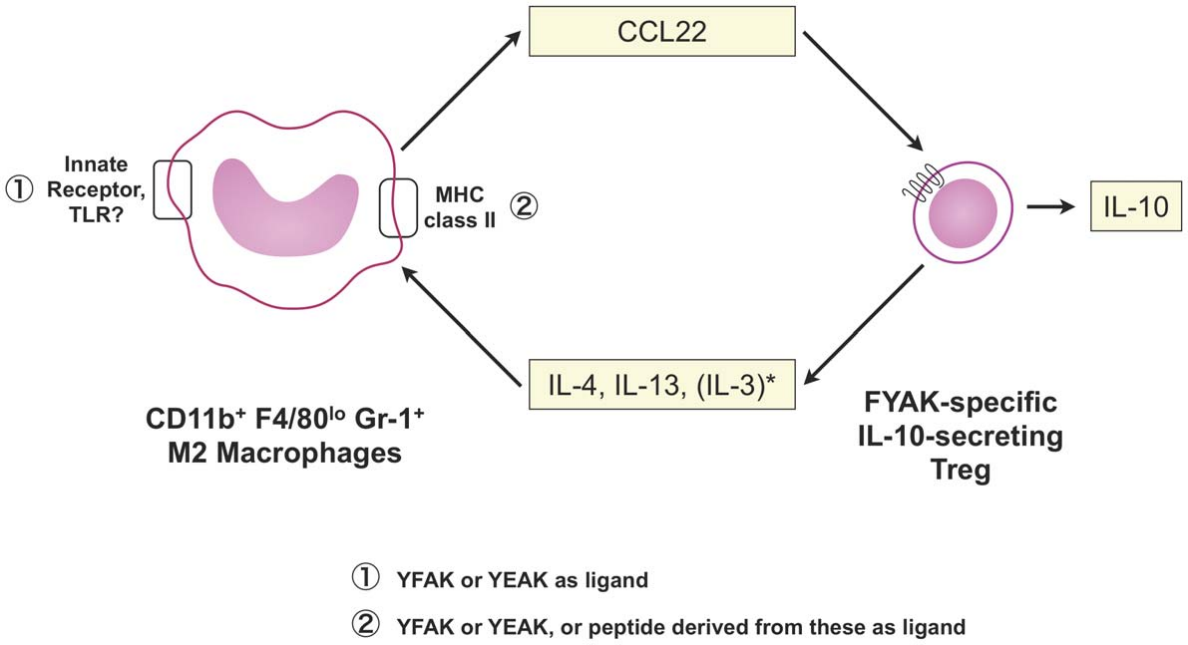
Hypothesis: Role of M2 macrophages in generation of IL-10 secreting T cells. It is not known whether IL-3 is secreted by the same T cells that secrete IL-4 and IL-13.

Myeloid progenitors differentiated to macrophages *in vitro* in the presence of IL-3, as well as the macrophage cell line RAW264.7, were also shown to secrete CCL22 in response to YFAK and to YEAK at high concentrations (Figures 3 and 4). Moreover, splenic T cells induced by YFAK also secrete IL-3 (Figure 5), as well as the M2 macrophage differentiating cytokines IL 4 and IL-13. IL-3 is a cytokine that stimulates development of multiple lineages of hematopoietic cells including myeloid macrophages and neutrophils, but excluding lymphoid cells. These data lead to the hypothesis summarized in Figure 6 that an important role of regulatory macrophages stimulated by amino acid copolymers is to secrete the chemokine CCL22 that attracts T cells, leading to increased numbers of IL-10-secreting T cells. Whether this phenomenon results from expansion of a small, preexisting pool of these cells or whether the regulatory macrophages induce naïve T cells to differentiate into IL-10-secreting T cells remains a subject for further study. In turn the T cells secrete IL-13, IL-4, and IL-3 that stimulate the proliferation and differentiation of M2 macrophages.

The induction of these Tregs by YEAK [24], [25] and YFAK [4], [6], [26] are both well documented, and the latter have been extensively characterized. YFAK programs bone marrow progenitors to induce a population of CD11b^+^/GR-1^high^/F4/80^low^ macrophages, called myeloid suppressors, that can modulate an autoimmune response (Figures S1, S2). This phenotype for myeloid suppressor cells has been reported previously [19], [27], [28].

Some differences in cytokine/chemokine production were observed between cells cultured in the presence of YFAK or YEAK (Figure 4). YFAK significantly increased supernatant concentrations of CCL22 (MDC) and of CXCL13 (BCA-1). CCL22 and CXCL13 are chemokines produced by M2 macrophages which are important in the homeostasis of lymphocyte trafficking and are a chemoattractant for T cells and B cells, respectively [15], [29]. CCL9 (MIP-1γ), an important chemokine that attracts CD11b^+^ DC and myeloid cells [30], was also increased as a result of YFAK treatment (Figure S4). Thus, CCL9 may play a role in the attraction/induction of myeloid suppressor cells which have been shown to be important in decreasing disease severity [31].

Some data suggest that CCL22 and CXCL13 may be produced by different cell populations. The serum level of CXCL13 induced *in vivo* by YFAK was greater than that of CCL22 (Figures 1 and 2). However, *in vitro* the level of CCL22 induced from myeloid progenitors was 50–100 times that of CXCL13, and YEAK did not induce CXCL13 from these cells in a detectable amount (Figure 4). Finally, although both YFAK and YEAK induced CCL22 secretion from RAW 264.7 murine macrophages (Figure 3), neither copolymer induced CXCL13 from these cells.

In addition, YFAK significantly decreased the secretion of the chemokines CXCL1 and CXCL2 and of pro-inflammatory cytokines TNF-α, IL-6, and IL-12p70. The pro-inflammatory properties associated with TNF-α play a major role in autoimmune diseases and interference with TNF- α production is a major treatment modality [32]. Etanercept (Enbrel®), infliximab (Remicade®), and adalimumab (Humira®) are TNF inhibitors that are used in animal disease models of MS [33] and have been approved for treatment of patients with rheumatoid arthritis, inflammatory bowel disease, and psoriasis [34]. IL-12p70 has also been shown to drive a T_H_1 immune response [35] and decreasing IL-12p70 production can modulate the immune response towards a T_H_2 immune response [36]. Since modulation of pro-inflammatory cytokines is associated with current MS treatment [37], this effect of YFAK could directly relate to its efficacy in EAE. By contrast, YEAK had little or no effect on secretion of these chemokines and cytokines in various assays.

The relationship between dose of YFAK and efficacy in EAE is of considerable interest, i.e., 0.75 mg/kg is inefficient at generating efficacy, 2.5 mg/kg gives significant efficacy, and at 7.5 mg/kg efficacy is lost. As the dose of YFAK increased, so did activation of the innate immune response (Figure 4). However, in vitro CCL22 secretion reached a peak of 6 µM and then declined. YEAK, which is ineffective at low concentration, peaked at 12 µM. If higher concentrations of YEAK had been used, a decline in CCL22 secretion may also have been seen. A decline in CCL22 secretion at high YFAK concentrations was also seen in an additional *in vitro* assay (N.K., unpublished observation), and was paralleled in Phase I clinical trials in secondary progressive MS patients by increased serum concentrations of CCL22, IL-3, and IL-13 at low dosages followed by decreases at higher dosages [9]. However, as the dose of either copolymer increased, a progressive loss in IL-3 production from T cells occurred (Figure 5). T cell activity alone is not solely responsible for efficacy because YFAK and YEAK would both have similar efficacy and the lower the dose the higher the T cell activity, i.e., 0.25 mg/kg had more IL-3 production than 2.5 mg/kg. However, 2.5 mg/kg is more efficacious for therapy. The innate immune response is also not solely responsible for efficacy. Together these data imply that both the innate (macrophages) and adaptive (T cells) immune responses are responsible for efficacy.

Interaction between cells of the innate and adaptive immune system is quite important in the mechanism. Th17 cells are believed to play a major role in autoimmune pathologies and multiple sclerosis in particular. YEAK has been shown to dampen differentiation of Th17 cells through altered production of IL-6 by monocytes [38]. YFAK reduces IL-6 secretion more severely (Figure 4) and may effectively control the expansion and differentiation of Th17 cells. IL-13, a cytokine produced in large quantity by T cells activated with both YEAK and YFAK, has a major effect on the differentiation of monocytes [39]. Moreover, production of IL-13 by these T cells could directly or indirectly (through antigen-presenting cells) affect the expansion of Th17 cells, since it is known that the IL-13 receptor is expressed by Th17 cells and that IL-13 attenuates Th17 cytokine production [40]. Thus, the optimal dosage requirement for YFAK is one that strongly stimulates the innate response, yet is low enough to not abrogate an adaptive immune response.

In summary, chemokine release from myeloid cells, which occurs rapidly on administration of amino acid copolymers *in vivo*, has significant implications for the systemic effect of these therapeutic agents on both innate and adaptive immunity. The differential effects of YFAK and YEAK on the innate immune system may explain the enhanced efficacy of YFAK in the treatment of EAE and is also likely to lead to better clinical efficacy.

## Supporting Information

Figure S1 **CD11b and F4/80 myeloid cell phenotypes induced from murine bone marrow by YFAK or YEAK.** Flow cytometric analysis was performed to identify phenotypic populations. (A–E) Data shown represents gating of CD11b versus F4/80 cell populations. (A–C) Data shown is a representative sample from cell populations administered 6.0 µM copolymer or medium control. (D and E) In vitro copolymer concentrations administered were 1.5, 3.0, and 6.0 µM. Samples were run in quadruplicate and are shown as percentage of cells ± SEM. Significance was calculated using an unpaired t-test: YFAK vs. YEAK * p≤0.05, *** p≤0.001. Medium control and PLP 139–151 produced similar results.
(DOCX)

Figure S2 **CD11b and Gr-1 Myeloid cell phenotypes induced by YFAK or YEAK.** Flow cytometric analysis was performed to identify phenotypic populations. (A–E) Data shown represents gating of CD11b versus Gr-1 cell populations. (A–C) Data shown is a representative sample from cell populations administered 6.0 µM copolymer or medium control. (D and E) In vitro copolymer concentrations administered were 1.5, 3.0, and 6.0 µM. Samples were run in quadruplicate and are shown as percentage of cells ± SEM. Significance was calculated using an unpaired t-test: YFAK vs. YEAK * p≤0.05, ** p≤0.01, *** p≤0.001. Medium control and PLP 139–151 produced similar results.
(DOCX)

Figure S3 **Disease progression after daily administration of YFAK, YEAK, or Vehicle.** Female SJL mice were induced to develop EAE as described in previous publications. Mice received daily administrations s.c. of 2.5 mg/kg YFAK, 2.5 mg/kg YEAK, or Vehicle beginning after the onset of disease. Mean ± SEM of disease progression from initial signs of disease is shown. Since treatment started after the onset of disease, the graph shows disease progression in relation to the initial level of disease (baseline disease) for each mouse. Significance was calculated using a Mann-Whitney t-test: YFAK vs. YEAK * p≤0.05.
(DOCX)

Figure S4 **CCL9 (MIP-1γ) concentration in culture supernatant of bone marrow-derived macrophages stimulated by copolymers.**
(DOC)

Table S1 **Soluble Factors tested using RBM Rodent MAP 1.6.** The data obtained that are not shown in the text are available upon request.
(DOCX)

## References

[1] Teitelbaum D, Meshorer A, Hirshfeld T, Arnon R, Sela M (1971). Suppression of experimental allergic encephalomyelitis by a synthetic polypeptide.. Eur J Immunol.

[2] Teitelbaum D, Webb C, Bree M, Meshorer A, Arnon R, Sela M (2005). Suppression of experimental allergic encephalomyelitis by a synthetic polypeptide.. Eur J Immunol.

[3] Fridkis-Hareli M, Santambrogio L, Stern JN, Fugger L, Brosnan C (2002). Novel synthetic amino acid copolymers that inhibit autoantigen-specific T cell responses and suppress experimental autoimmune encephalomyelitis.. J Clin Invest.

[4] Stern JN, Illés Z, Reddy J, Keskin DB, Sheu E (2004). Amelioration of proteolipid protein 139–151-induced encephalomyelitis in SJL mice by modified amino acid copolymers and their mechanisms.. Proc Natl Acad Sci USA.

[5] Illés Z, Stern JN, Reddy J, Waldner H, Mycko MP (2004). Modified amino acid copolymers suppress myelin basic protein 85–99- induced encephalomyelitis in humanized mice through different effects on T cells.. Proc Natl Acad Sci USA.

[6] Stern JN, Keskin DB, Zhang H, Lv H, Kato Z (2008). Amino acid copolymer-specific IL-10-secreting regulatory T cells that ameliorate autoimmune diseases in mice.. Proc Natl Acad Sci USA.

[7] Yin H, Vistica BP, Chan CC, Strominger JL, Gery I (2009). Inhibition of experimental autoimmune uveitis by amino acid copolymers.. J Neuroimmunol.

[8] Kovalchin J, Krieger J, Collins K, Genova M, Augustyniak M (2011). Safety, Pharmacokinetic, and Pharmacodynamic Evaluations of PI-2301, a Potent Immunomodulator, in a First-in-Human, Single-Ascending-Dose Study in Healthy Volunteers.. J Clinical Pharma.

[9] Kovalchin J, Krieger J, Genova M, Collins K, Augustyniak M (2010). Results of a phase I study in patients suffering from secondary-progressive multiple sclerosis demonstrating the safety of the amino acid copolymer PI-2301 and a possible induction of an anti-inflammatory cytokine response.. J Neuroimmunol.

[10] Hussien Y, Sanna A, Söderström M, Link H, Huang YM (2001). Glatiramer acetate and IFN-beta act on dendritic cells in multiple sclerosis.. J Neuroimmunol.

[11] Vieira PL, Heystek HC, Wormmeester J, Wierenga EA, Kapsenberg ML (2003). Glatiramer Acetate (Copolymer-1, Copaxone) Promotes Th2 Cell Development and Increased IL-10 Production Through Modulation of Dendritic Cells.. J Immunol.

[12] Kim HJ, Ifergan I, Antel JP, Seguin R, Duddy M (2004). Type 2 Monocyte and Microglia Differentiation Mediated by Glatiramer Acetate Therapy in Patients with Multiple Sclerosis.. J Immunology.

[13] Weber MS, Prod'homme T, Youssef S, Dunn SE, Rundle CD (2007). Type II monocytes modulate T cell-mediated central nervous system autoimmune disease.. Nat Med.

[14] Kala M, Rhodes SN, Piao WH, Shi FD, Campagnolo DI (2010). B cells from glatiramer acetate-treated mice suppress experimental autoimmune encephalomyelitis.. Exp Neurol.

[15] Mantovani A, Sica A, Sozzani S, Allavena P, Vecchi A (2004). The chemokine system in diverse forms of macrophage activation and polarization.. Trends Immunol.

[16] Fridkis-Hareli M, Teitelbaum D, Gurevich E, Pecht I, Brautbar C (1994). Direct binding of myelin basic protein and synthetic copolymer 1 to class II major histocompatibility complex molecules on living antigen-presenting cells–specificity and promiscuity.. Proc Natl Acad Sci USA.

[17] Fridkis-Hareli M, Strominger JL (1998). Promiscuous binding of synthetic copolymer 1 to purified HLA-DR molecules.. J Immunol.

[18] Kuroda E, Ho V, Ruschmann J, Antignano F, Hamilton M (2009). SHIP represses the generation of IL-3-induced M2 macrophages by inhibiting IL-4 production from basophils.. J Immunol.

[19] Makarenkova VP, Bansal V, Matta BM, Perez LA, Ochoa JB (2006). CD11b+/Gr-1+ myeloid suppressor cells cause T cell dysfunction after traumatic stress.. J Immunol.

[20] Li Q, Milo R, Panitch H, Swoveland P, Bever CT (1998). Glatiramer acetate blocks the activation of THP-1 cells by interferon-gamma.. Eur J Pharmacol.

[21] Hohlfeld R, Wekerle H (2004). Autoimmune concepts of multiple sclerosis as a basis for selective immunotherapy: from pipe dreams to (therapeutic) pipelines.. Proc Natl Acad Sci.

[22] Racke MK, Lovett-Racke AE, Karandikar NJ (2010). The mechanism of action of glatiramer acetate treatment in multiple sclerosis.. Neurology.

[23] Gordon S, Martinez FO (2010). Alternative activation of macrophages: mechanism and functions.. Immunity.

[24] Jee Y, Piao WH, Liu R, Bai XF, Rhodes S (2007). CD4(+)CD25(+) regulatory T cells contribute to the therapeutic effects of glatiramer acetate in experimental autoimmune encephalomyelitis.. Clin Immunol.

[25] Venken K, Hellings N, Broekmans T, Hensen K, Rummens JL (2008). Natural naïve CD4+CD25+CD127low regulatory T cell (Treg) development and function are disturbed in multiple sclerosis patients: recovery of memory Treg homeostasis during disease progression.. J Immunol.

[26] Zhang H, Stern JN, Strominger JL (2009). T cell receptors in an IL-10-secreting amino acid copolymer-specific regulatory T cell line that mediates bystander immunosuppression.. Proc Natl Acad Sci USA.

[27] Zhu B, Bando Y, Xiao S, Yang K, Anderson AC (2007). CD11b^+^Ly-6C^hi^ suppressive monocytes in experimental autoimmune encephalomyelitis.. J Immunol.

[28] Gabrilovich DI, Nagaraj S (2009). Myeloid-derived suppressor cells as regulators of the immune system.. Nat Rev Immunol.

[29] Mantovani A, Sozzanic S, Locati M, Allavena P, Sica A (2002). Macrophage polarization: tumor-associated macrophages as a paradigm for polarized M2 mononuclear phagocytes.. Trends in Immunology.

[30] Mohamadzadeh M, Poltorak AN, Bergstressor PR, Beutler B, Takashima A (1996). Dendritic cells produce macrophage inflammatory protein-1 gamma, a new member of the CC chemokine family.. J Immunol.

[31] Sinha P, Clements VK, Ostrand-Rosenberg S (2005). Reduction of myeloid-derived suppressor cells and induction of M1 macrophages facilitate the rejection of established metastatic disease.. J Immunol.

[32] Aggarwal BB, Shishodia S, Takada Y, Jackson-Bernitsas D, Ahn KS (2006). TNF blockade: an inflammatory issue.. Ernst Schering Res Found Workshop.

[33] Glabinksi AR, Bielecki B, Kawczak JA, Tuohy VK, Selmaj K (2004). Treatment with soluble tumor necrosis factor receptor (sTNFR):Fc/p80 fusion protein amerliorates relapsing-remitting experimental autoimmune encephalomyelitis and decreases chemokine expression.. Autoimmunity.

[34] Sethi G, Sung B, Kunnumakkara AB, Aggarwal BB (2009). Targeting TNF for treatment of cancer and autoimmunity.. Adv Exp Med Biol.

[35] Gee K, Guzzo C, Che Mat NF, Ma W, Kumar A (2009). The IL-12 family of cytokines in infection, inflammation and autoimmune disorders.. Inflamm Allergy Drug Targets.

[36] Wu C, Yang G, Bermúdez-Humarán LG, Pang Q, Zeng Y (2006). Immunomodulatory effects of IL-12 secreted by *Lactococcus lactis* on Th1/Th2 balance in ovalbumin (OVA)-induced asthma model mice.. International Immunopharmacol 2006;.

[37] Mellergård J, Edström M, Vrethem M, Ernerudh J, Dahle C (2010). Natalizumab treatment in multiple sclerosis: marked decline of chemokines and cytokines in cerebrospinal fluid.. Mult Scler.

[38] Chen C, Liu X, Wan B, Zhang JZ (2009). Regulatory properties of copolymer I in Th17 differentiation by altering STAT3 phosphorylation.. J Immunol.

[39] Scotton CJ, Martinez FO, Smelt MJ, Sironi M, Locati M (2005). Transcriptional profiling reveals complex regulation of the monocyte IL-1 beta system by IL-13.. J Immunol.

[40] Newcomb DC, Zhou W, Moore ML, Goleniewska K, Hershey GK (2009). A functional IL-13 receptor is expressed on polarized murine CD4+ Th17 cells and IL-13 signaling attenuates Th17 cytokine production.. J Immunol.

