# Supplementary figures and images for: Macrophage-Specific Chemokines Induced via Innate Immunity by Amino Acid Copolymers and Their Role in EAE
Source: PLoS One. 2011 Dec 15;6(12):e26274. doi: 10.1371/journal.pone.0026274 (PMC3240613; doi:10.1371/journal.pone.0026274)

**Supplemental Figure 1**

**
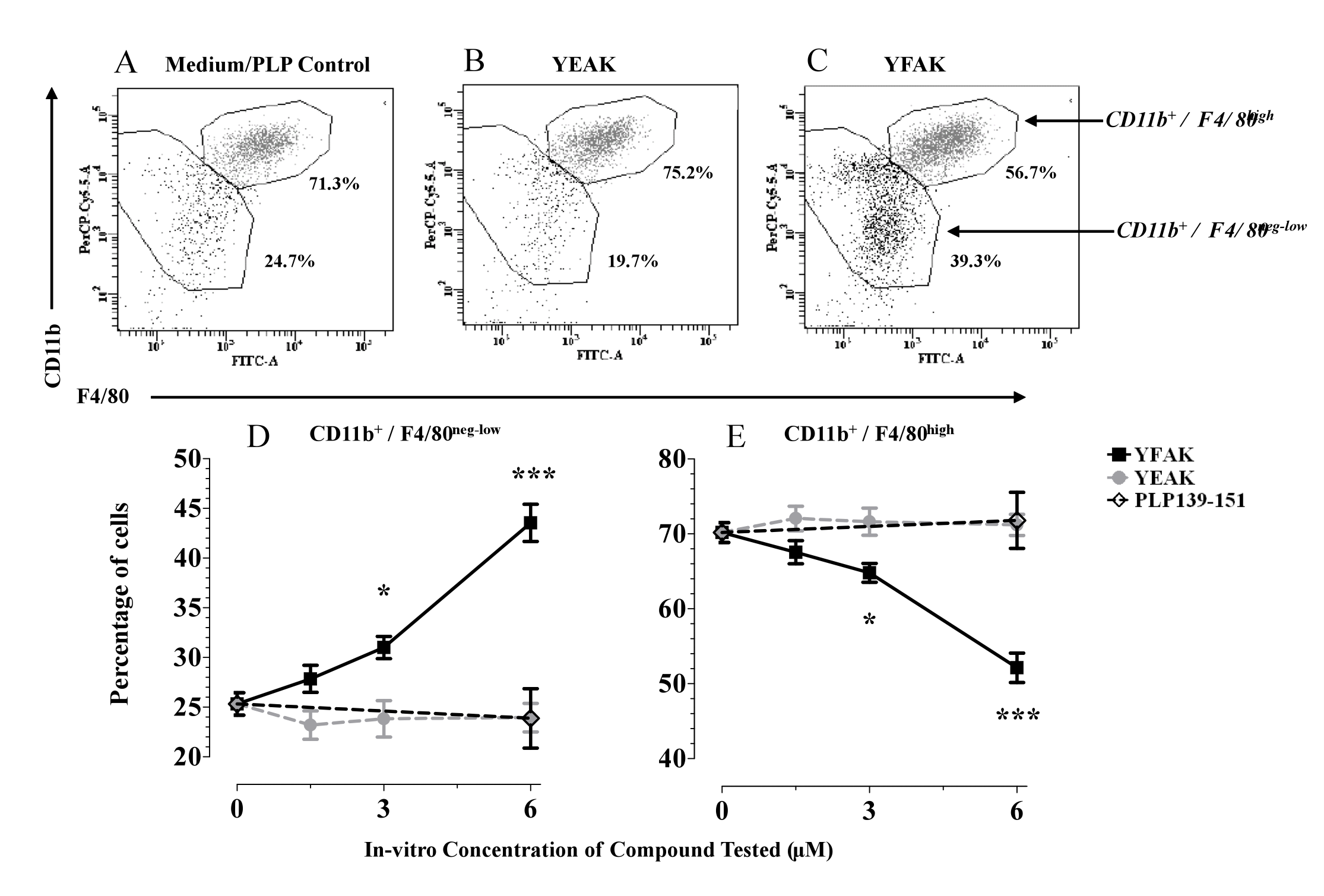
**

Supplement: Figure S1 — CD11b and F4/80 myeloid cell phenotypes induced from murine bone marrow by YFAK or YEAK. Flow cytometric analysis was performed to identify phenotypic populations. (A–E) Data shown represents gating of CD11b versus F4/80 cell populations. (A–C) Data shown is a representative sample from cell populations administered 6.0 µM copolymer or medium control. (D and E) In vitro copolymer concentrations administered were 1.5, 3.0, and 6.0 µM. Samples were run in quadruplicate and are shown as percentage of cells ± SEM. Significance was calculated using an unpaired t-test: YFAK vs. YEAK * p≤0.05, *** p≤0.001. Medium control and PLP 139–151 produced similar results. (DOCX) [file pone.0026274.s001.docx]

**Supplemental Figure 2**

**
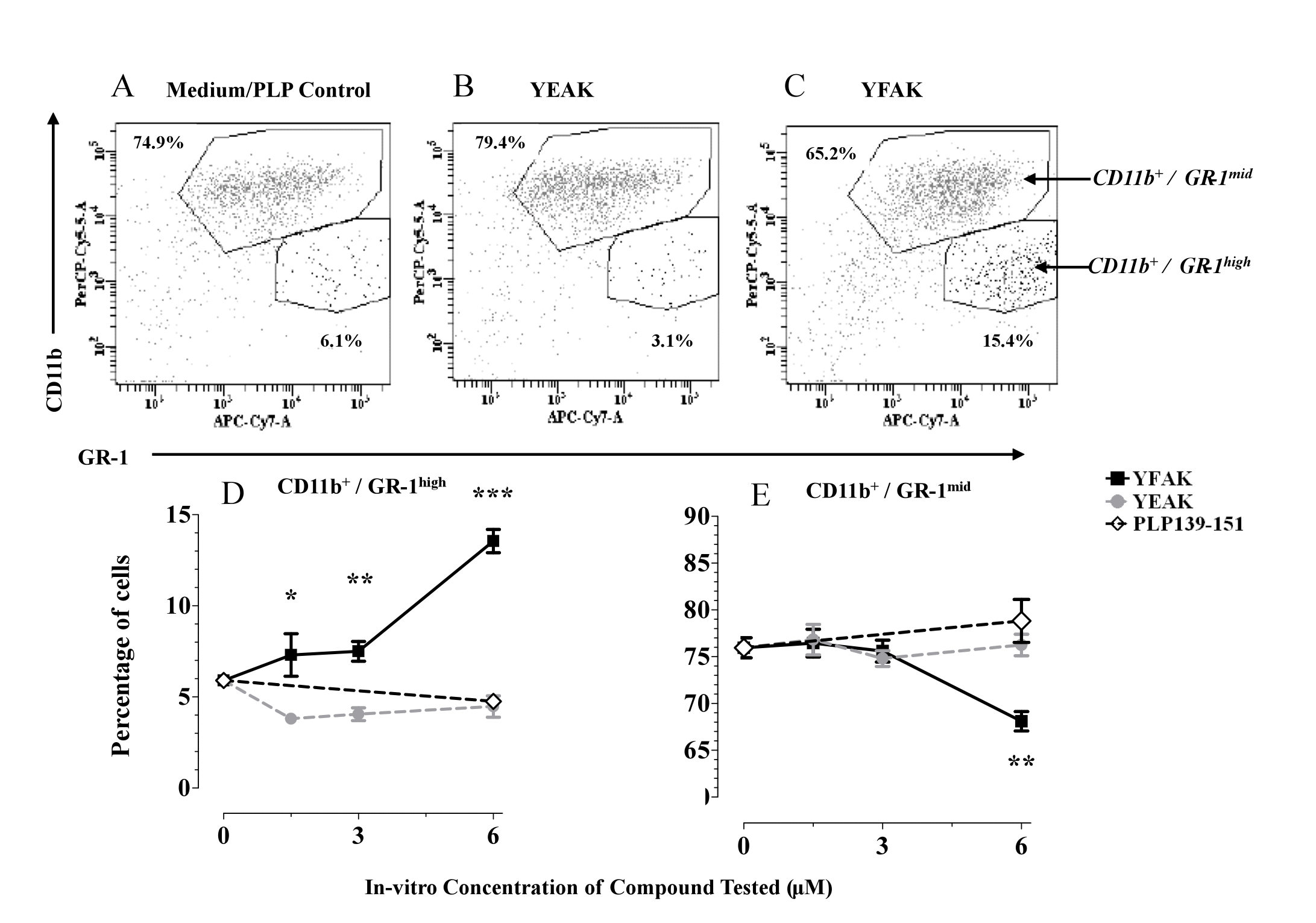
**

Supplement: Figure S2 — CD11b and Gr-1 Myeloid cell phenotypes induced by YFAK or YEAK. Flow cytometric analysis was performed to identify phenotypic populations. (A–E) Data shown represents gating of CD11b versus Gr-1 cell populations. (A–C) Data shown is a representative sample from cell populations administered 6.0 µM copolymer or medium control. (D and E) In vitro copolymer concentrations administered were 1.5, 3.0, and 6.0 µM. Samples were run in quadruplicate and are shown as percentage of cells ± SEM. Significance was calculated using an unpaired t-test: YFAK vs. YEAK * p≤0.05, ** p≤0.01, *** p≤0.001. Medium control and PLP 139–151 produced similar results. (DOCX) [file pone.0026274.s002.docx]

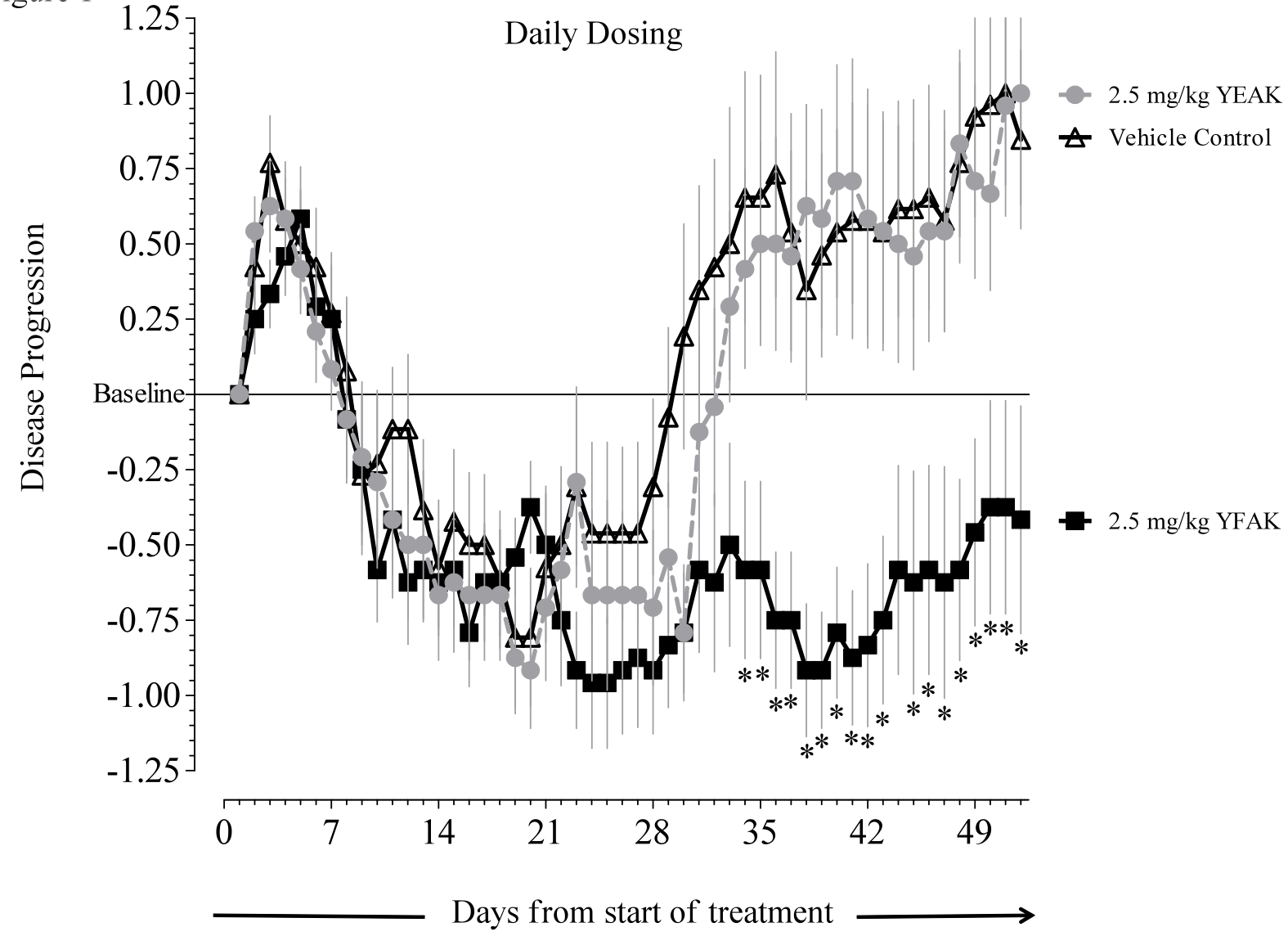


**Supplemental Figure 3**

Supplement: Figure S3 — Disease progression after daily administration of YFAK, YEAK, or Vehicle. Female SJL mice were induced to develop EAE as described in previous publications. Mice received daily administrations s.c. of 2.5 mg/kg YFAK, 2.5 mg/kg YEAK, or Vehicle beginning after the onset of disease. Mean ± SEM of disease progression from initial signs of disease is shown. Since treatment started after the onset of disease, the graph shows disease progression in relation to the initial level of disease (baseline disease) for each mouse. Significance was calculated using a Mann-Whitney t-test: YFAK vs. YEAK * p≤0.05. (DOCX) [file pone.0026274.s003.docx]

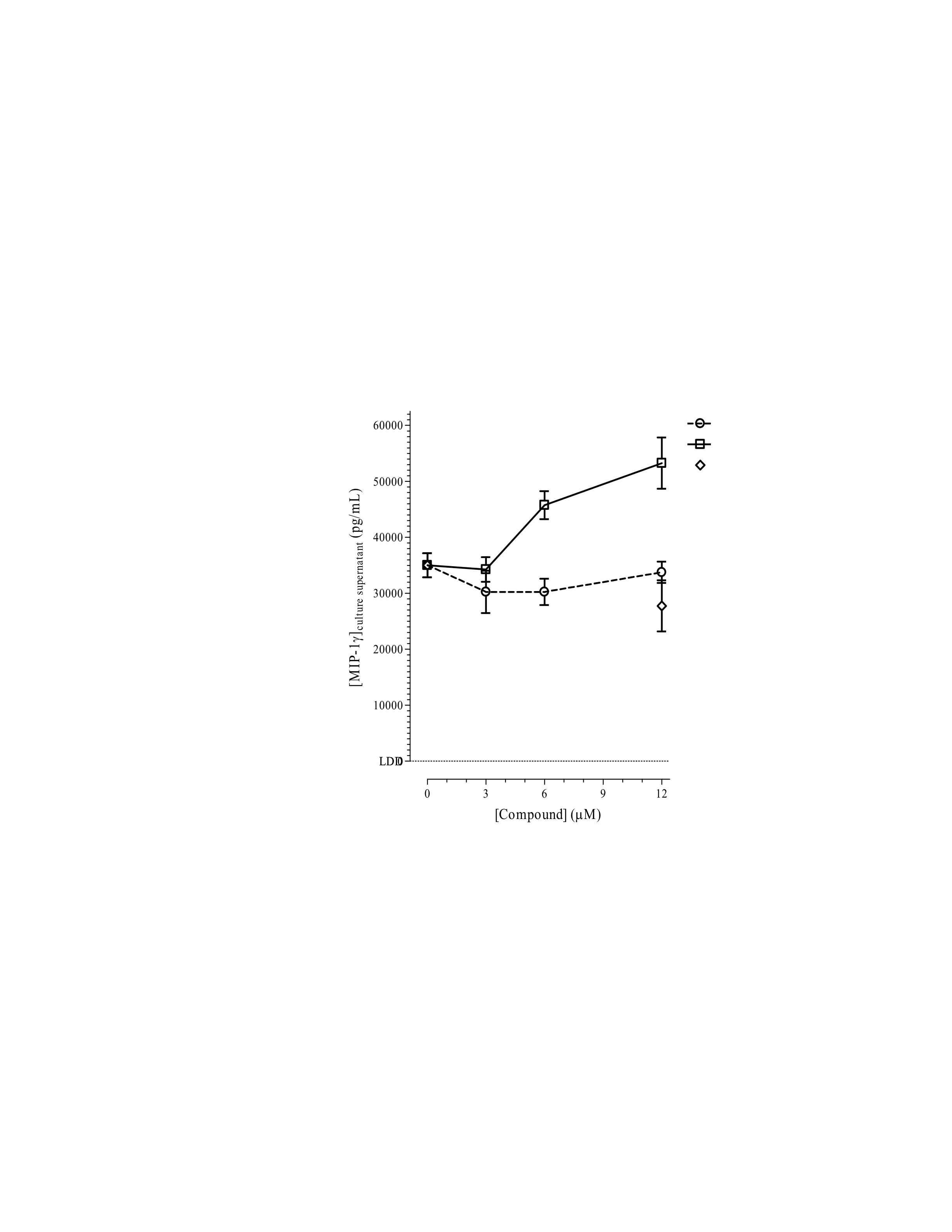


**Supplemental Figure 4**

YEAK

YFAK

Control

Supplement: Figure S4 — CCL9 (MIP-1γ) concentration in culture supernatant of bone marrow-derived macrophages stimulated by copolymers. (DOC) [file pone.0026274.s004.doc]

| ***Chemokines, Cytokines and Soluble Factors Tested in RBM Rodent Map 1.6*** | | |
| --- | --- | --- |
| 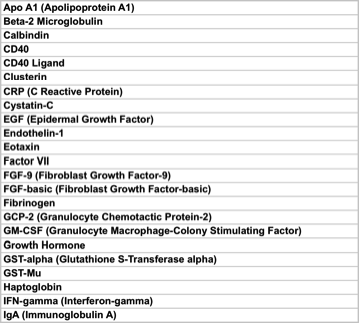 | 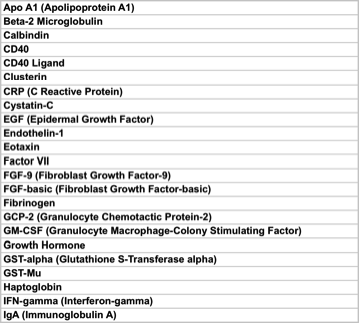 | 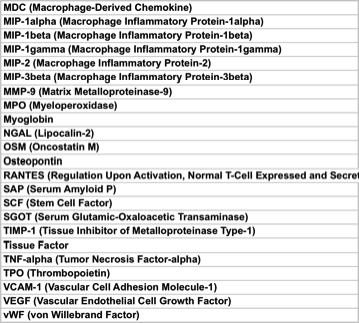 |

Supplement: Table S1 — Soluble Factors tested using RBM Rodent MAP 1.6. The data obtained that are not shown in the text are available upon request. (DOCX) [file pone.0026274.s005.docx]
